# Functional Abnormalities of the Endocrine System in Beta-Thalassemia Major Patients: Insights From a Hospital-Based Observational Study

**DOI:** 10.7759/cureus.100190

**Published:** 2025-12-27

**Authors:** Upendra Prasad Sahu, Yuthika Kumari, Neha Rani, Omar Hasan, Naghma Mobin, Shrasta Soumya, Praveen Kumar Singh, Neetu Kumari, Niraj Kumar

**Affiliations:** 1 Department of Pediatrics, Rajendra Institute of Medical Sciences, Ranchi, IND

**Keywords:** beta-thalassemia major, blood transfusion, chelation therapy, diabetes mellitus, endocrine dysfunction, growth hormone deficiency, hypoparathyroidism, hypothyroidism, iron overload, pediatric hematology

## Abstract

Background

Beta-thalassemia major (BTM) predisposes children to several endocrine complications, primarily driven by iron overload and repeated transfusions. This study aimed to determine the prevalence, patterns, and risk factors of endocrine dysfunction among multi-transfused pediatric patients with BTM.

Methods

A hospital-based cross-sectional study was conducted at Rajendra Institute of Medical Sciences (RIMS), Ranchi, including 100 pediatric patients aged two to 14 years diagnosed with BTM. Clinical profiles, transfusion history, anthropometric parameters, and laboratory investigations, including HbA1c, thyroid profile, serum ferritin, ALP, calcium, phosphate, parathyroid hormone, and cortisol, were analyzed. Statistical tests were performed using IBM SPSS Statistics version 26, with p ≤ 0.05 considered significant.

Results

Endocrine disorders were highly prevalent: diabetes mellitus (14.6%), growth hormone deficiency (11.2%), hypothyroidism (9.8%), and hypoparathyroidism (6.3%). Mean HbA1c was 6.8 ± 1.2%, with significantly higher levels in patients with diabetes (p = 0.002), hypothyroidism (p = 0.035), and growth hormone deficiency (p = 0.018). Logistic regression showed that iron overload (p = 0.022), frequent transfusions (p = 0.059), and older age (>10 years) (p = 0.041) were major predictors of endocrine dysfunction, while chelation therapy did not show a strong protective effect (p = 0.072).

Conclusion

Multi-transfused BTM patients are at substantial risk of developing endocrine abnormalities due to cumulative iron overload and chronic transfusion exposure. Regular endocrine screening, optimized iron chelation, and individualized transfusion protocols are essential to prevent long-term metabolic and hormonal complications. Strengthening multidisciplinary care is crucial for improving growth, development, and overall outcomes in this vulnerable population.

## Introduction

Beta-thalassemia major (BTM) is a severe hereditary anemia caused by defective beta-globin synthesis, leading to lifelong transfusion dependence and chronic iron overload. Despite improved survival with modern transfusion and chelation therapy, significant long-term complications persist. Among these, endocrine disorders are particularly common due to iron deposition in the pituitary, thyroid, pancreas, adrenal glands, and gonads [1-4]. The resulting hormonal dysfunction leads to growth retardation, hypothyroidism, diabetes mellitus, hypogonadism, and adrenal insufficiency, all of which negatively impact growth, metabolism, and reproductive health [5-7]. Early recognition and appropriate management of these complications are essential to improving the quality of life and long-term prognosis in patients with BTM [8].

The prevalence and severity of endocrine dysfunction in BTM vary across populations and are influenced by transfusion frequency, chelation efficacy, and genetic background [9]. In many developing regions, these complications remain underrecognized due to limited diagnostic resources and inadequate routine screening [10-11]. This hospital-based observational study aims to determine the prevalence and spectrum of endocrine disorders in BTM patients, identify associated risk factors, and emphasize the need for routine endocrine evaluation. The findings are expected to support early diagnosis, guide timely interventions, and contribute to strategies that improve long-term outcomes and quality of life in this vulnerable population [12].

## Materials and methods

Study design and setting

This hospital-based cross-sectional observational study was conducted from March 2024 to February 2025 in the Department of Pediatrics, Rajendra Institute of Medical Sciences (RIMS), Ranchi, Jharkhand, India.

Participants

The study included pediatric patients aged two to 14 years diagnosed with beta thalassemia major, who attended the Department of Pediatrics, RIMS, Ranchi, Jharkhand, during the study period. Patients were eligible if the diagnosis was confirmed by hemoglobin electrophoresis and if they had complete laboratory data, including complete blood count (CBC) to assess hematological status, glycosylated hemoglobin (HbA1c) to evaluate long-term glycemic control, thyroid profile, serum calcium and phosphate, alkaline phosphatase (ALP), parathyroid hormone (PTH), and serum ferritin, along with a detailed clinical history and thorough physical examination. Both male and female patients on regular follow-up at the department were included. Patients with other hemolytic anemias, such as alpha thalassemia, sickle cell anemia, or hereditary spherocytosis, were excluded.

Sample size calculation

The sample size for this study was calculated using the standard formula for estimating a population proportion:

  n=(z^2^×P×Q)/d^2^ =(〖1.96〗^2^×0.07×0.93)/〖0.05〗^2^ )= 100.16,

where:

n = required sample size

Z = Z-score corresponding to the desired confidence level

P = estimated prevalence of the condition

Q = 1 − P

d = relative allowable error

This formula was used to ensure statistical validity and representativeness. Based on these calculations, the required sample size was estimated to be 100 patients. Accordingly, 100 pediatric BTM patients meeting the inclusion criteria were randomly selected and enrolled in the study.

Data collection and laboratory assays

Blood samples were collected from each participant using a standardized protocol. Two samples were drawn with an 8-mL syringe and transferred into EDTA tubes. After obtaining informed consent, samples were analyzed in the Department of Laboratory Medicine, RIMS, Ranchi. Laboratory investigations included CBC, HbA1C, serum ferritin, thyroid profile (T3, T4, TSH), serum calcium, phosphate, ALP, and parathyroid hormone (PTH).

Random blood sugar (RBS), growth hormone (GH) stimulation tests, insulin-like growth factor-1 (IGF-1), adrenocorticotropic hormone (ACTH), and cortisol stimulation tests were not routinely performed due to limited availability, high cost, and lack of standardized dynamic testing facilities in the study setting. Although baseline serum cortisol was measured, dynamic adrenal function testing could not be undertaken. Endocrine dysfunction was therefore assessed primarily through clinical evaluation, auxological parameters, and available biochemical markers, reflecting real-world constraints in a tertiary government hospital.

Growth hormone deficiency was diagnosed based on clinical growth failure, height-for-age below −2 SD, reduced growth velocity, and delayed bone age, rather than dynamic GH stimulation testing. Diabetes mellitus was diagnosed using HbA1c ≥6.5% or previously documented diabetes on treatment. Hypothyroidism was defined by elevated TSH with low or normal free T4. Hypoparathyroidism was diagnosed based on low serum calcium with inappropriately low or normal PTH levels.

All laboratory analyses were performed using standardized methods following institutional quality-control protocols.

Statistical analysis

A sample size of 100 patients was calculated based on an estimated prevalence of 7%, a 95% confidence interval, and a relative allowable error of 5%. Data were collected using a pre-tested, semi-structured questionnaire covering sociodemographic variables, parental consanguinity, family history of endocrine disorders, duration of illness, anthropometric parameters, iron chelation therapy patterns, and frequency of blood transfusions. Laboratory findings related to endocrine assessments were also recorded.

Data were tabulated using Microsoft Excel (Microsoft Corp., USA) and analyzed with IBM SPSS Statistics for Windows, Version 26.0 (released 2018, IBM Corp., Armonk, NY). Both parametric and non-parametric tests were applied based on the normality of data distribution, and a p-value ≤ 0.05 was considered statistically significant. Statistical analyses were performed to determine the prevalence and associations of endocrine dysfunction among patients with beta thalassemia major.

Ethical considerations

Approval for the study was granted by the Institutional Ethics Committee of RIMS, Ranchi (Memo No. 76-IEC-RIMS, dated 20.02.2024). Written parental consent and age-appropriate child assent were obtained prior to enrollment.

## Results

Demographic distribution and anthropometric characteristics

A total of 100 pediatric BTM patients were included in the study, of whom 64% were males and 36% were females, indicating a clear male predominance (Table 1). Age distribution showed that most patients were in late childhood to early adolescence, with 28% aged 0-5 years, 37% aged 6-10 years, and 35% aged 11-14 years (Table 2). Weight assessment revealed that the majority (79%) fell within the 11-30 kg range, with 5% weighing 0-10 kg, 37% weighing 11-20 kg, 42% weighing 21-30 kg, 19% weighing 31-40 kg, and 1% weighing 41-50 kg (Table 3).

**Table 1:** Gender-wise distribution of the respondents

Gender (n = 100)	Frequency	Percentage (%)
Female	36	36%
Male	64	64%

**Table 2:** Age-wise distribution of the respondents

Age distribution (years)	Frequency	Percentage (%)
0-5 years	28	28%
6-10 years	37	37%
11-14 years	35	35%
Total: 100 patients	Total = 100	Total = 100%

**Table 3:** Weight-wise distribution of the respondents

Weight distribution (kg)	Frequency	Percentage (%)
0-10	5	5%
11-20	37	37%
21-30	42	42%
31-40	19	19%
41-50	01	01%

Anthropometric assessment included weight and age-appropriate interpretation. Weight was analyzed in relation to age and clinical context to reflect nutritional and growth status. Although formal z-scores could not be calculated due to incomplete height data in some patients, the predominance of low body weight supports the presence of chronic growth impairment commonly observed in BTM.

The mean weight of the cohort was 22.87 kg (SD 8.14), indicating considerable variability likely associated with chronic anemia and growth impairment.

Biochemical and hormonal parameters of the study cohort

Biochemical analysis revealed considerable variability across multiple metabolic and hormonal parameters. ALP levels (Table 4) ranged from 60 to 410 U/L, with a mean of 186.34 U/L and a standard deviation of 74.13 U/L, indicating marked differences in bone and hepatic activity among patients. HbA1c levels (Table 5) showed a wide distribution, ranging from 4.5% to 14.5%, with a mean of 6.8%, median of 6.7%, SD of 1.2, and an IQR of 1.6, reflecting considerable variability in glycemic control. The number of blood transfusions (Table 6) also varied, with a mean of 6.13, a median of 6, an SD of 2.42, and a range of 2 to 10 transfusions per month. PTH levels (Table 7) ranged from 35 to 85 pg/mL, with a mean of 58.27 pg/mL, a median of 57.50 pg/mL, and an SD of 14.62, demonstrating notable fluctuation in parathyroid function. Cortisol levels (Table 8) showed moderate variability, ranging from 10.50 to 27.30 µg/dL, with a mean of 18.42 µg/dL, a median of 18.30 µg/dL, and an SD of 4.76 µg/dL.

**Table 4:** Alkaline phosphatase (ALP) level distribution (U/L)

Serial no.	Parameter	Value (U/L)
01	Minimum ALP	60
02	Maximum ALP	410
03	Mean ALP	186.34
04	Standard deviation	74.13

**Table 5:** HbA1c distribution (%)

Serial no.	Statistic value	Values (%)
01	Mean	6.8
02	Median	6.7
03	Standard deviation	1.2
04	Minimum	4.5
05	Maximum	14.5
06	Interquartile range (IQR)	1.6

**Table 6:** Monthly transfusion frequency distribution

Statistic	Value
Mean	6.13
Median	6.00
Standard deviation	2.42
Minimum	2
Maximum	10

**Table 7:** Parathyroid hormone (PTH) level descriptive values

Statistic	Value (Pg/ mL)
Mean	58.27
Median	57.50
Standard deviation	14.62
Minimum	35.00
Maximum	85.00

**Table 8:** Cortisol level distribution

Statistic	Value (µg/dL)
Mean	18.42
Median	18.30
Standard deviation	4.76
Minimum	10.50
Maximum	27.30

Prevalence and distribution of endocrine disorders

Endocrine disorders were frequently observed in the study population. In the present study, endocrine dysfunction was identified in both symptomatic and asymptomatic patients. Several endocrine abnormalities were detected during routine biochemical screening before overt clinical manifestations became evident, indicating a substantial burden of subclinical endocrine dysfunction. Symptomatic patients presented with features such as poor growth velocity, weight faltering, or glycemic abnormalities, whereas asymptomatic patients were diagnosed based on abnormal laboratory parameters during scheduled follow-up evaluations. This underscores the importance of routine endocrine surveillance in BTM patients, as reliance solely on clinical symptoms may delay diagnosis and intervention.

**Figure 1:**
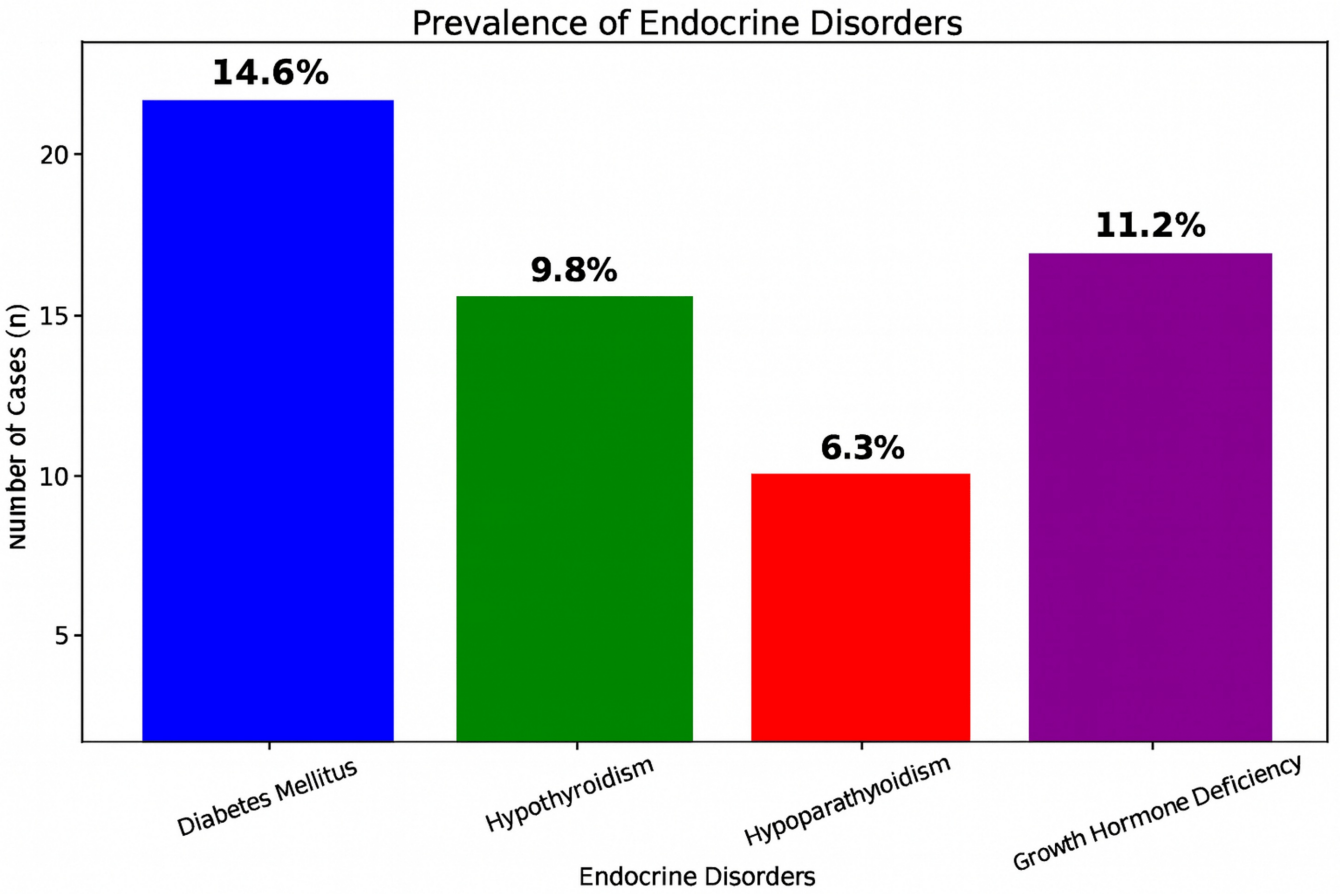
Prevalence of endocrine disorders in beta-thalassemia major patients (n = 100)

**Figure 2:**
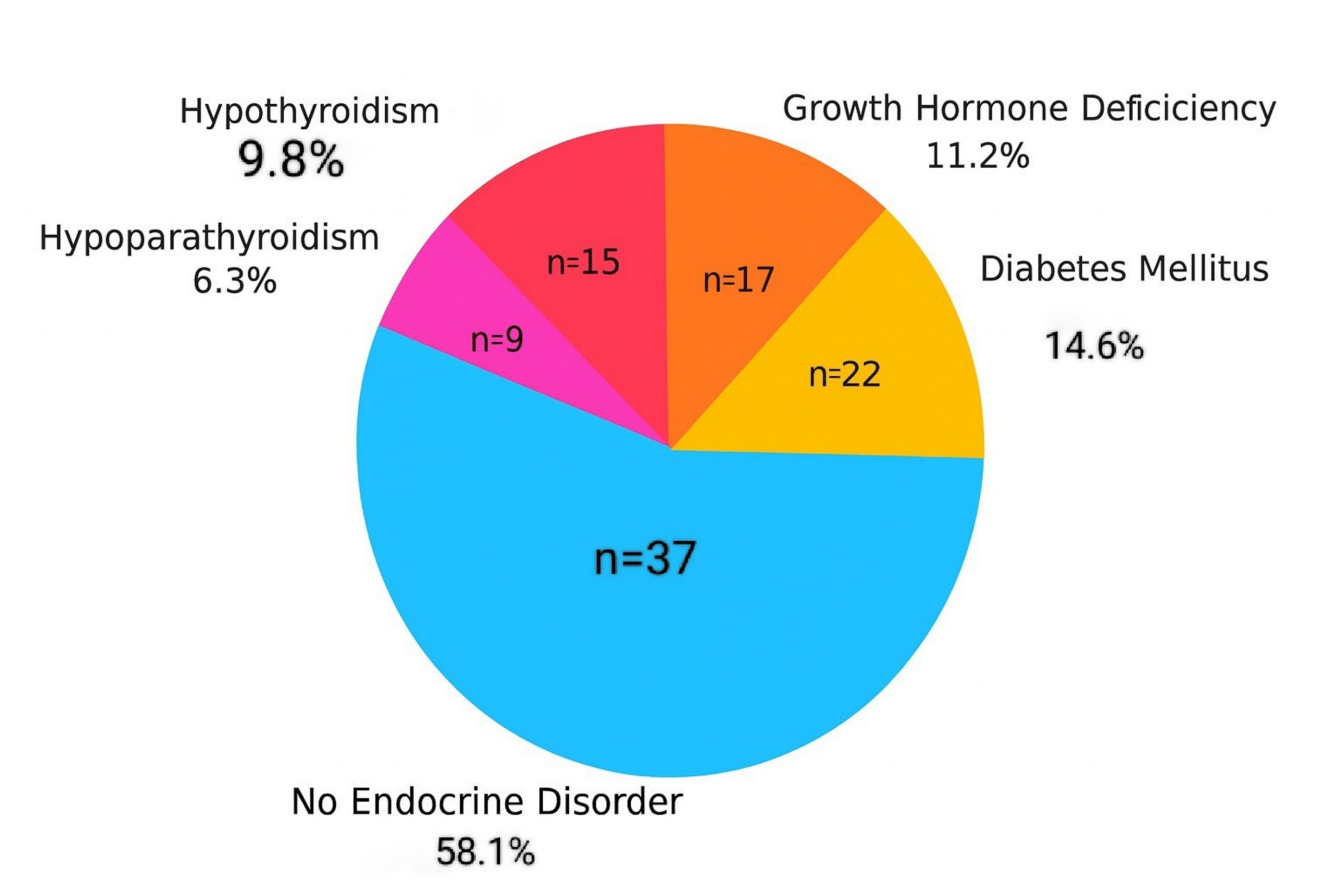
Prevalence of endocrine dysfunction in beta-thalassemia major patients (n = 100)

The prevalence of endocrine abnormalities included diabetes mellitus (14.6%), growth hormone deficiency (11.2%), hypothyroidism (9.8%), and hypoparathyroidism (6.3%), confirming a substantial endocrine burden among multi-transfused patients. HbA1c levels were significantly elevated in patients with diabetes (p = 0.002), hypothyroidism (p = 0.035), and growth hormone deficiency (p = 0.018), indicating a strong association between these conditions and impaired glycemic regulation. Although hypoparathyroidism was associated with a mild HbA1c increase, the difference was not statistically significant (p = 0.068) (Table 9, Figures 1-2).

**Table 9:** Prevalence of endocrine disorders in beta-thalassemia major patients

Endocrine disorder	Number of cases (n)	Percentage (%)
Diabetes mellitus	22	14.6%
Growth hormone deficiency	17	11.2%
Hypothyroidism	15	9.8%
Hypoparathyroidism	9	6.3%

Association of clinical and transfusion-related factors with endocrine dysfunction

Logistic regression analysis identified iron overload, transfusion frequency, and age as significant predictors of endocrine dysfunction among BTM patients. Patients with serum ferritin levels >1000 ng/mL had a significantly higher risk of developing endocrine abnormalities (OR 3.2, p = 0.022). Those receiving ≥3 transfusions per month demonstrated a markedly higher prevalence of endocrine dysfunction (70.6%) compared to patients receiving fewer transfusions (38%), showing borderline statistical significance (p = 0.059). Older age (>10 years) was also significantly associated with endocrine dysfunction (p = 0.041), reflecting the cumulative impact of prolonged transfusion exposure and iron toxicity. Chelation therapy did not demonstrate a statistically significant protective effect (p = 0.072), suggesting that iron-related endocrine injury may persist despite treatment.

Furthermore, endocrine dysfunction showed a progressive increase with advancing age, rising serum ferritin levels, and increasing transfusion burden. Patients aged >10 years exhibited a substantially higher prevalence of endocrine abnormalities compared to younger children. Similarly, the prevalence of endocrine disorders increased markedly with escalating iron overload, reaching its highest levels among patients with serum ferritin >5000 ng/mL. A higher cumulative transfusion burden (≥100 units) was also associated with significantly increased endocrine morbidity. These trends were consistently observed across all endocrine disorders studied, including diabetes mellitus, hypothyroidism, hypoparathyroidism, and growth hormone deficiency, underscoring the central role of iron toxicity and transfusion intensity in the pathogenesis of endocrine dysfunction in BTM patients (Table 10).

**Table 10:** Association of age, transfusion burden, and serum ferritin with endocrine dysfunction

Category	Variable	Patients (n)	Endocrine dysfunction
Age (years)	≤10 years	48	18 (37.5%)
	>10 years	52	39 (75.0%)
Serum ferritin level (ng/ml)	<2500 ng/ml	30	8 (26.7%)
	2501–5000 ng/ml	34	18 (52.9%)
	>5000 ng/ml	36	31 (86.1%)
Blood transfusion (unit/yr)	<100 units/yr	41	14 (34.1%)
	≥100 units/yr	59	43 (72.9%)

Overall, the results demonstrate a high prevalence of endocrine abnormalities in BTM patients and identify iron overload, transfusion burden, and older age as major contributing factors, emphasizing the need for routine endocrine monitoring and optimized iron control.

## Discussion

The present study highlights a significant burden of endocrine dysfunction among multi-transfused BTM patients, strongly linked to chronic transfusions and iron overload. The developing pediatric population is particularly vulnerable due to increased iron uptake in metabolically active organs and ongoing endocrine maturation, which magnifies susceptibility to iron-induced hormonal injury. In community-based pediatric cohorts, the prevalence of diabetes mellitus is typically below 1%, and hypothyroidism occurs in approximately 1-2% of children, while growth hormone deficiency and hypoparathyroidism are rare conditions [13]. By contrast, the present study identified markedly higher rates of diabetes mellitus (14.6%), hypothyroidism (9.8%), growth hormone deficiency (11.2%), and hypoparathyroidism (6.3%) among BTM patients. This disparity highlights the disease-specific endocrine vulnerability associated with chronic transfusion therapy and iron overload. The significantly higher prevalence observed in this cohort underscores the need for routine endocrine surveillance in thalassemia patients, as reliance on population-based screening thresholds may lead to delayed diagnosis and missed early intervention opportunities [14-18].

HbA1c variability, with markedly higher levels in patients with diabetes, hypothyroidism, and growth hormone deficiency, confirms the impact of endocrine dysfunction on glucose regulation [19]. Although hypoparathyroidism demonstrated a mild HbA1c elevation, it was not statistically significant. However, even this mild rise is clinically relevant in thalassemia due to chronic hemolysis influencing HbA1c reliability. Other endocrine abnormalities contributed notably to impaired metabolic control [19]. Prolonged transfusion exposure leads to hemosiderin deposition in endocrine tissues such as the pituitary and pancreas, contributing to insulin deficiency and blunted hormonal secretion, which directly correlates with glucose dysregulation and growth-delay phenotypes. Logistic regression further identified iron overload, transfusion frequency, and older age as significant predictors of endocrine dysfunction. Pancreatic β-cell siderosis may precipitate an insulinopenic-diabetes phenotype, especially after 10 years of age, when cumulative transfusion burden rises sharply. Despite ongoing medical care, chelation therapy showed no statistically significant protective benefit, indicating persistent iron-related endocrine injury despite treatment [15, 20]. This suggests that prescription alone is insufficient, and therapeutic outcomes likely depend more on dose adequacy and adherence - parameters not always captured in cross-sectional design.

Patient characteristics, including the predominance of children aged six to 14 years, male majority, low body weight, and wide ALP variation, provide additional insight into disease impact [21]. Growth failure and pubertal delay in thalassemia are multifactorial, resulting from iron-induced pituitary siderosis, chronic anemia, nutritional deficits, and target-organ endocrine toxicity. Iron-induced pituitary dysfunction has been implicated in growth failure and delayed pubertal development in BTM, based on clinical manifestations rather than direct hormonal measurements in this study. The variability in pubertal progression across the cohort reinforces the heterogeneity of endocrine iron infiltration. Growth failure, delayed puberty, and abnormal glucose metabolism remain key complications and are established consequences of iron-mediated endocrine tissue injury. The observed variability in low body-weight Z-scores reflects the combined effects of chronic undernutrition, recurrent anemia, iron overload, and underlying endocrine dysfunction, highlighting the dynamic interaction between nutritional status and hormonal regulation in children with BTM. Altered bone metabolism - driven by iron toxicity and hormone insufficiency - contributes to skeletal morbidity. The broad range of ALP levels observed in this study supports a high burden of skeletal involvement in BTM, likely reflecting altered bone turnover [21-26].

Although serum calcium and vitamin D levels were not systematically assessed, elevated ALP may be attributable to a combination of bone remodeling abnormalities and hepatic involvement secondary to iron overload, as reported in previous studies. These parameters emphasize both bone and hepatic vulnerability in heavily transfused children. Taken together, these findings underscore the need for routine endocrine screening, early detection of hormonal abnormalities, optimized iron chelation, adherence-monitored therapy protocols, age-targeted surveillance, and individualized management plans. In resource-limited settings such as Jharkhand, serum ferritin remains a practical surrogate marker of iron overload; however, MRI T2* imaging of the liver, pancreas, and pituitary provides a non-invasive quantitative assessment of tissue iron deposition and could allow earlier detection of endocrine risk and more precise correlation with hormonal dysfunction in future studies. Family counseling and caregiver education on long-term chelation compliance should accompany every transfusion visit.

A coordinated multidisciplinary approach involving pediatric endocrinology, hematology, and nutrition services is recommended to prevent irreversible endocrine morbidity, enable early detection of subclinical dysfunction, and maintain normal growth and metabolic stability. In particular, early identification of growth failure suggestive of pituitary growth hormone suppression may help prevent progressive and irreversible short stature in patients with BTM. In addition to HbA1c, future studies should incorporate more sensitive glycemic assessments, such as the oral glucose tolerance test (OGTT), to better detect early glucose dysregulation. Optimizing the calcium-phosphate-vitamin D axis is also important for maintaining bone health and preventing metabolic bone disease. Timely evaluation and management of hypogonadotropic hypogonadism are essential to guide appropriate puberty-induction therapy and support normal pubertal development in affected children.

Such integration could reduce morbidity and significantly improve health-related quality of life, linear growth potential, metabolic health, school performance, and pubertal attainment, especially in multi-transfused children, who are likely to transition into endocrinopathy-risk adolescence soon. These outcomes remain consistent with iron-induced endocrine injury seen globally. Moreover, institutional screening policy updates informed by this study may serve as a regional care model. Chelation intensification protocols and iron adherence audits should be prioritised in future RIMS cohorts.

Limitations and future directions

This study has several limitations that should be acknowledged. Being a single-center, hospital-based cross-sectional study, the findings may have limited generalizability to the wider BTM population, and causal relationships between iron overload, transfusion burden, chelation therapy, and endocrine dysfunction cannot be established. The lack of longitudinal follow-up precludes assessment of the temporal progression of endocrine abnormalities and reversibility with optimized chelation. Advanced imaging modalities such as MRI T2* for direct quantification of iron deposition in endocrine organs (pancreas, pituitary, liver) were not available, necessitating reliance on serum ferritin as a surrogate marker, which may not fully reflect tissue iron burden. Dynamic endocrine testing, including GH stimulation tests, IGF-1, ACTH, and detailed gonadotropin and sex-steroid assays, could not be routinely performed due to resource limitations; therefore, some endocrine diagnoses were based on clinical and available biochemical parameters rather than gold-standard dynamic testing. Glycemic assessment relied primarily on HbA1c, which may underestimate dysglycemia in thalassemia due to shortened red cell lifespan, hemolysis, and frequent transfusions; alternative markers such as OGTT, fructosamine, or continuous glucose monitoring could provide greater diagnostic accuracy in future studies. Anthropometric assessment was limited by incomplete height data in some patients, preventing calculation of standardized z-scores, and bone health evaluation lacked systematic calcium-phosphate-vitamin D profiling in all participants. Finally, although chelation therapy status was recorded, detailed adherence assessment and dose-response analysis could not be performed, limiting the interpretation of its protective effect against endocrine complications

Future research should explore longitudinal follow-up to evaluate endocrine recovery following optimized chelation therapy, distinguishing reversible from irreversible glandular damage and identifying the most responsive developmental periods for intervention. Incorporating pancreatic T2* MRI alongside OGTT and insulin secretion indices may enable earlier detection of β-cell siderosis and improve prediction models for iron-induced diabetes. Furthermore, studies focusing on pubertal development, i.e., tracking Tanner staging, sex-hormone profiles, bone age, and psychosocial outcomes, are needed to better understand the impact of early endocrine intervention and guide puberty-induction therapy in affected adolescents.

## Conclusions

This study confirms a high prevalence of endocrine disorders, particularly diabetes mellitus, hypothyroidism, hypoparathyroidism, and growth hormone deficiency, among multi-transfused pediatric patients. BTM exhibits a high prevalence (42.1%) of endocrine dysfunction, predominantly driven by chronic iron overload (ferritin >1000 ng/mL), increasing age, and transfusion burden. Glycemic control was significantly altered in patients with diabetes, hypothyroidism, and growth hormone deficiency, as reflected by elevated HbA1c levels, while hypoparathyroidism showed only a mild, non-significant increase. The analysis demonstrates that iron overload is the strongest predictor of endocrine dysfunction, with frequent blood transfusions and older age also contributing significantly to hormonal abnormalities. These findings underscore that cumulative transfusion exposure and prolonged iron burden play a central role in the pathogenesis of endocrine complications in thalassemia.

Chelation therapy did not demonstrate a statistically significant protective effect against endocrine dysfunction in our cohort. This may reflect inconsistent adherence, delayed initiation, or suboptimal chelation intensity, highlighting the need for early initiation and strict long-term compliance rather than prescription alone. The results highlight the importance of routine endocrine screening, careful monitoring of glycemic control, individualized transfusion protocols, and early therapeutic intervention to prevent irreversible hormonal damage. Overall, this study emphasizes the necessity of comprehensive, multidisciplinary management to improve long-term metabolic, growth, and developmental outcomes in BTM patients, and supports the need for further research to optimize prevention and treatment strategies for endocrine complications in this population.
